# Identification of Immune-Related Genes Associated With Bladder Cancer Based on Immunological Characteristics and Their Correlation With the Prognosis

**DOI:** 10.3389/fgene.2021.763590

**Published:** 2021-11-26

**Authors:** Zhen Kang, Wei Li, Yan-Hong Yu, Meng Che, Mao-Lin Yang, Jin-Jun Len, Yue-Rong Wu, Jun-Feng Yang

**Affiliations:** ^1^ The Affiliated Hospital, Kunming University of Science and Technology, Kunming, China; ^2^ Department of Urology, The First People’s Hospital of Yunnan Province, Kunming, China

**Keywords:** bladder cancer, ssGSEA, tumor immunity, immune characteristics, urology

## Abstract

Background:To identify the immune-related genes of bladder cancer (BLCA) based on immunological characteristics and explore their correlation with the prognosis. Methods:We downloaded the gene and clinical data of BLCA from the Cancer Genome Atlas (TCGA) as the training group, and obtained immune-related genes from the Immport database. We downloaded GSE31684 and GSE39281 from the Gene Expression Omnibus (GEO) as the external validation group. R (version 4.0.5) and Perl were used to analyze all data. Result:Univariate Cox regression analysis and Lasso regression analysis revealed that 9 prognosis-related immunity genes (PIMGs) of differentially expressed immune genes (DEIGs) were significantly associated with the survival of BLCA patients (*p* < 0.01), of which 5 genes, including *NPR2, PDGFRA, VIM, RBP1, RBP1* and *TNC*, increased the risk of the prognosis, while the rest, including *CD3D, GNLY, LCK,* and *ZAP70,* decreased the risk of the prognosis. Then, we used these genes to establish a prognostic model. We drew receiver operator characteristic (ROC) curves in the training group, and estimated the area under the curve (AUC) of 1-, 3- and 5-year survival for this model, which were 0.688, 0.719, and 0.706, respectively. The accuracy of the prognostic model was verified by the calibration chart. Combining clinical factors, we established a nomogram. The ROC curve in the external validation group showed that the nomogram had a good predictive ability for the survival rate, with a high accuracy, and the AUC values of 1-, 3-, and 5-year survival were 0.744, 0.770, and 0.782, respectively. The calibration chart indicated that the nomogram performed similarly with the ideal model. Conclusion:We had identified nine genes, including *PDGFRA, VIM, RBP1, RBP1*, *TNC*, *CD3D, GNLY, LCK,* and *ZAP70*, which played important roles in the occurrence and development of BLCA. The prognostic model based on these genes had good accuracy in predicting the OS of patients and might be promising candidates of therapeutic targets. This study may provide a new insight for the diagnosis, treatment and prognosis of BLCA from the perspective of immunology. However, further experimental studies are necessary to reveal the underlying mechanisms by which these genes mediate the progression of BLCA.

## Introduction

Bladder cancer (BLCA) is one of the 10 most common cancers around the world, with 550,000 new cases and 200,000 deaths in 2018 (Richters et al., 2020). The risk of BLCA is 1 in 74 for men and 1 in 301 for women, and in the past decade, the number of new cases of BLCA has increased by 32% (Fitzmaurice et al., 2019). As we all know, non-muscle invasive bladder cancer (NMIBC) and muscle invasive bladder cancer (MIBC) are the two main types of bladder cancer. When patients progress from NMIBC to MIBC, their overall survival (OS) rate significantly decreases (Cao et al., 2020a; Tran et al., 2021), and about one-third of NMIBC patients will develop MIBC (Sylvester et al., 2006). As we all know, bladder cancer diagnosis represents a challenge for clinicians, and currently available diagnostic and staging tools include: 1) urine cytological analysis; 2) cystoscopy and pathological biopsy; 3) computed tomography or magnetic resonance imaging. However, all of the above-mentioned tools have some defects, such as low sensitivity or demands for invasive operation (van Rhijn et al., 2009). Tumor markers, as a new research tool, can not only help clinicians understand the characteristics of tumors, but also help early diagnosis, improve prognosis and carry out risk stratification and targeted therapy for tumor patients (Bratu et al., 2021). So far, there have been many studies on blood (Dohn et al., 2021), tissue and urine markers (Aibara et al., 2021; Chen et al., 2021; Tosev et al., 2021) of bladder cancer, and clinical guidelines are paying more attention to the application of clinical tumor markers (Witjes et al., 2021). Especially, genetic testing often performs better in predicting the prognosis, and multi-gene prognostic models are gradually becoming the choice of more clinicians (Qu et al., 2021).

In recent years, immune checkpoint inhibitors (ICPIs) have revolutionized the treatment paradigm for most malignant tumors with persistent positive responses even observed in advanced and refractory cancers (Bindal et al., 2021). Therefore, exploring the interaction between tumor cells and immunity can help clinicians gain a deeper understanding of the occurrence, development and metastasis of BLCA (Guan et al., 2021). So far, a lot of recent studies have performed the analysis of the immune characteristics of BLCA patients, which have fully demonstrated that immune genes have higher predictive values of the prognosis, and provide better clinical guidance than routine clinical features or risk models (Cao et al., 2020b; Wang et al., 2021; Zhang et al., 2021). However, these studies only evaluated the immunological characteristics of BLCA from the view of immune cell infiltration, and lacked the exploration on the tumor-immune interaction and its potential values of predicting the prognosis of BLCA.

The tumor microenvironment (TME) consists of immune cells, stromal cells, extracellular vesicles and other molecules. A study showed that TME was an important regulator of gene expression and was closely involved in the occurrence, development and treatment of tumors (Kumari et al., 2021). The immune system and immune response play a crucial role in TME (Dzobo, 2020). In this study, we innovatively used single-sample gene set enrichment analysis (ssGSEA) to classify BLCA patients into a high-immune (Immunity_H) group and a low-immune (Immunity_L) group, and then explored the tumor-immune interaction, related molecular characteristics, and the potential prognosis from the perspective of immune-difference-related genes. Finally, we used these genes and the machine learning method of the Least Absolute Shrinkage and Selection Operator (Lasso) algorithm to establish a prognostic model, and validated the stability and repeatability of the model in an external independent data set.

## Materials and Methods

### Data Collection

The Cancer Genome Atlas (TCGA) (https://portal.gdc.cancer.gov/) is a landmark cancer genomics program that molecularly describes over 20,000 primary cancer, and matches normal samples spanning 33 cancer types. This joint effort between National Cancer Institute (NCI) and the National Human Genome Research Institute began in 2006, and has produced over 2.5 petabytes of genomic, epigenomic, transcriptomic, and proteomic data. The data, which has already led to improvements in our ability to diagnose, treat, and prevent cancer, will remain publicly available for anyone in the research community to use. We downloaded FPKM standardized RNA-seq data, clinical information and tumor mutation burden (TMB) information from the TCGA-BLCA cohort in TCGA database.

ImmPort (https://www.immport.org/) is funded by the National Institute of Health (NIH) and National Institute of Allergy and Infectious Diseases (NIAID) in support of the NIH mission to share data with the public. We clicked the “Resources” button on the Immport database homepage, then clicked the “Gene Lists” button on the “Resources” page, and finally clicked the “Gene Summary” to download immune-related genes.

Gene Expression Omnibus (GEO) is a public functional genomics data repository supporting MIAME-compliant data submissions. Array- and sequence-based data are accepted. Tools are provided to help users query and download experiments and curated gene expression profiles. We downloaded two data sets (GSE31684 and GSE39281) recording bladder cancer transcriptome genes (RNA-seq) and clinical information in the GEO database. After processing the data with Perl, we obtained two gene expression matrices. Then, we used the “sva” package in the R language (version 4.0.5) to merges the two expression matrices and eliminate batch effects.

### Data Analysis

(A) TMB analysis: we used BLCA mutation data in the TCGA database and Perl language to calculate the number of base mutations in each BLCA sample. (B) Single-sample gene set enrichment analysis (ssGSEA) and hierarchical cluster analysis: we used R packages (GSVA, GSEABase and limma) to perform ssGSEA to calculate the immune score of each BLCA sample according to 29 immune gene sets composed of different types of immune cells with different functions, pathways and checkpoints (Alhamdoosh et al., 2017). Firstly, the rank of gene expression values in a given BLCA sample was normalized, and then the enrichment score (ES) was calculated using the empirical cumulative distribution function. Each ssGSEA score XI was converted to XI′ by bias normalization to obtain the scores of different immune cells and immune-related functions in each sample. Then, we used the hierarchical clustering method of Euclidean distance and Ward linkage to do the immune stratification of BLCA patients. Meanwhile, we also made use of the T-distribution stochastic neighbor embedding (tSNE) algorithm to determine the immune stratification of BLCA patients through RtSEN package (Gardner et al., 2021). (C) Evaluation of tumor immune microenvironment: based on ESTIMATE algorithm, BLCA transcriptome data was utilized to predict stromal cell score, immune cell score and tumor purity, and then the content of these two types of cells was predicted, from which StromalScore, ImmuneScore and EstimateScore were determined (Yoshihara et al., 2013). (D) Tumor-infiltrating immune cells analysis: CIBERSORT, an R tool, was used for the deconvolution of the expression matrix of human immune cell subtypes according to linear support vector regression. This method is based on a known reference set and provides a set of gene expression characteristics of 22 immune cell subtypes. Therefore, we used the CIBERSORT method to do the calculation for the abundance of infiltrating immune cells in BLCA samples (Newman et al., 2015). (E) Immune differential genes determining the immune stratification: the limma package was utilized to select differentially expressed genes (DEGs) among people with different immune stratification (| log2 fold change | > 1.50 and FDR <0.05), and then we obtained immune-related genes from ImmPort (Bhattacharya et al., 2014). DEIGs were obtained through the intersection of immune genes and DEGs. (E) Prognostic markers: the survival package was utilized to do the univariate Cox regression analysis (*p* < 0.05) to identify the markers of significant prognosis-related immunity genes (PIMGs).

### Gene Set Pathway Enrichment Analysis

Gene set enrichment analysis was performed via the GSEA software (version 4.1.0) to analyze TCGA-BLCA transcriptomes for the identification of the key signaling pathways involved in DEGs.

The major Kyoto Encyclopedia of Genes and Genomes (KEGG) pathways involved in the up-regulation of the Immunity_H and Immunity_L subgroups (*p* < 0.05, FDR <0.01) were selected. R (version 4.0.5) was used to perform further analysis, and visualize the results. Then, we obtained transcription factors associated with the occurrence and development of bladder cancer from the CISTROME project, extracted differentially expressed transcription factors (DETFs) from the total DEGs, and used Pearson correlation coefficient analysis to construct the regulatory network of PIMGs and DETFs (R > 0.3 and FDR <0.01) (Mei et al., 2017). Finally, the protein-protein interaction (PPI) network analysis was performed using STRING (String-db.org/).

### Constructing and Validating the Prognostic Model of the Immune-Related Genes

We used the LASSO Cox regression model in R package (Dalal et al., 2012) “glmnet” to find genes significantly associated with the prognosis to construct the prognostic model of BLCA (PMB). The risk score was calculated as the following formula: $riskScore=\sum_{i=1}^{9}\beta_{i}*LPIMG_{i}$ , where LPIMG_i_ represented the i-th LPIMG (Lasso-prognosis-related immunity genes), and ***β***_i_ represented the expression coefficient of LPIMG_i_ obtained from Lasso regression analysis. All cases were classified into a low-risk group and a high-risk group based on the median risk score, and we performed the Kaplan-Meier survival analysis to compare the survival status between the high-risk group and the low-risk group. In order to verify the predictive power of PMB, the receiver operator characteristic (ROC) curve was drawn to calculate the area under the curve (AUC) of 1-, 3-, and 5-year survival. We conducted Kaplan-meier, logarithmic rank, ROC curve and calibration analysis using “timeROC,” “rms,” “survival,” and “survminer” software packages in R language. Based on the risk score calculated by PMB, Pearson correlation coefficient, Spearman correlation coefficient and corrplot package were used to evaluate the correlation between the risk score and overall survival, immune cell infiltration, immune checkpoint molecules and TMB. *p* < 0.05 of the critical value for the significant correlation was set. Eventually, univariate and multivariate Cox regression analysis of the risk scores of the constructed PMB and patients’ clinical characteristics (age, sex, stage) was performed to verify the accuracy of the independence of PMB-based risk characteristics. Based on the above factors, we created a nomogram using the R packages of “rms”, “nomogramEX” and “regplot.” Finally, the ROC and calibration chart were drew to determine the suitability of our established nomogram for potential clinical applications.

## Results

### Identification of Two Subtypes of BLCA Using Immune Analysis

**FIGURE 1:**
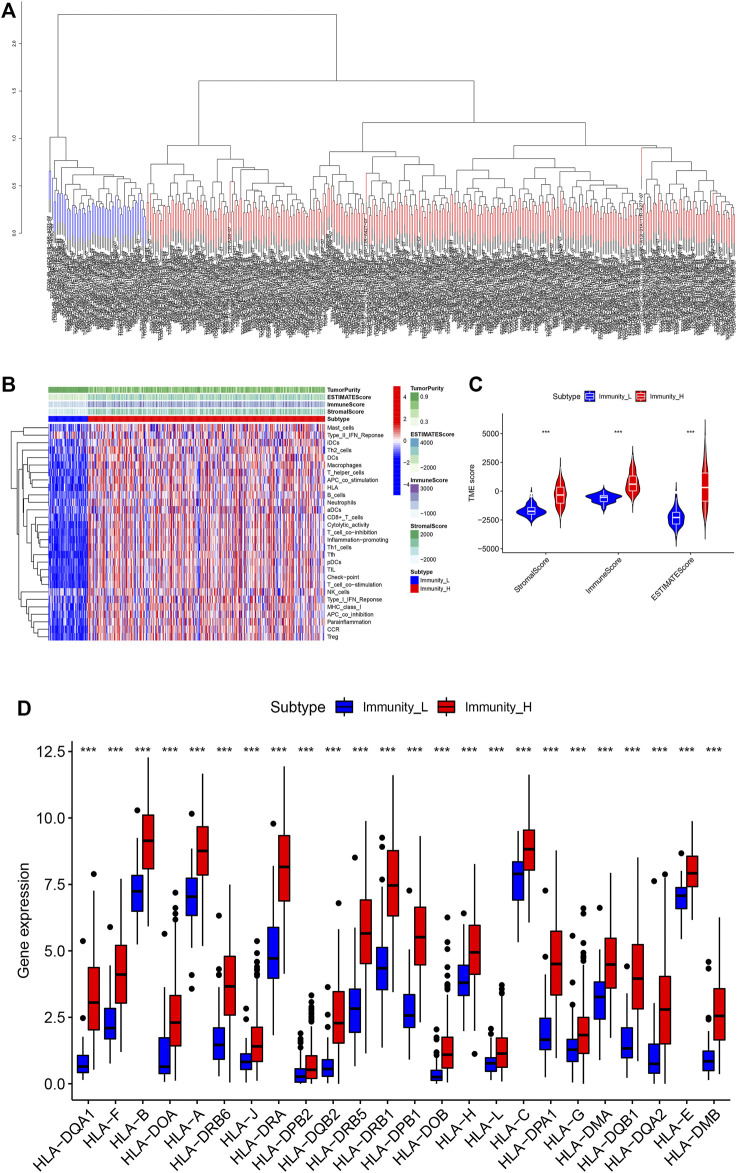
**(A)** The two immune types of BLCA patients, the red part was the high immune group, the blue was the low immune group. **(B)** The status of immune infiltration and tumor microenvironment (TME) in the TCGA-BLCA cases. **(C)** The comparisons of StromalScore, ESTIMATEScore, and ImmuneScore between the two subtypes. **(D)** The comparison of expression level of HLA gene between the two subtypes. *p < 0.05, **p < 0.01, ***p < 0.001.

In order to fully evaluate the immunological characteristics of BLCA, we used the ssGSEA to analyze 414 tumor samples from the TCGA-BLCA cohort. According to the ssGSEA scores and hierarchical clustering method, BLCA cases were divided into two clusters. The average score of the immune microenvironment of the first cluster was 0.62, and the average score of the immune microenvironment of the second cluster was 0.49. Thus, the first cluster was set as the Immunity_H (high) group, and the second cluster as the Immunity_L (low) group (Figures 1A,B). The tSNE was further used to analyze the immune levels for different BLCA patients and the same classification was obtained (Supplementary Figure S1A). The results of ESTIMATE analysis indicated that EstimateScore (419.27 ± 1649.47), ImmuneScore (750.39 ± 886.17), and StromalScore (-331.12 ± 910.28) in the Immunity_H group were significantly higher than those which were (−2283.37 ± 727.70), (−620.62 ± 352.59), and (−1662.75 ± 487.21), respectively, in the Immunity_L group (Wilcox test, *p* < 0.001) (Figure 1C). CIBERSORT was used to detect the degree of immune cell infiltration in the tumor, which found that the differences between the Immunity_H group and the Immunity_L group in T cells CD4 naive, T cells CD4 memory resting, T cells CD4 memory activated, NK cells resting, NK cells activated, Macrophages M1 and Mast cells activated were significant (Supplementary Figure S1B). The expression of human leukocyte antigen (HLA) genes in the both groups was examined, which suggested that most of HLA genes significantly increased in the Immunity_H group and significantly decreased in the Immunity_L group (Wilcox test, *p* < 0.05) (Figure 1D). Based on our results, we believed that immune response might play important roles in the development of BLCA.

Identification of immune-related genes associated with bladder cancer and their correlation with prognosis.

**FIGURE 2:**
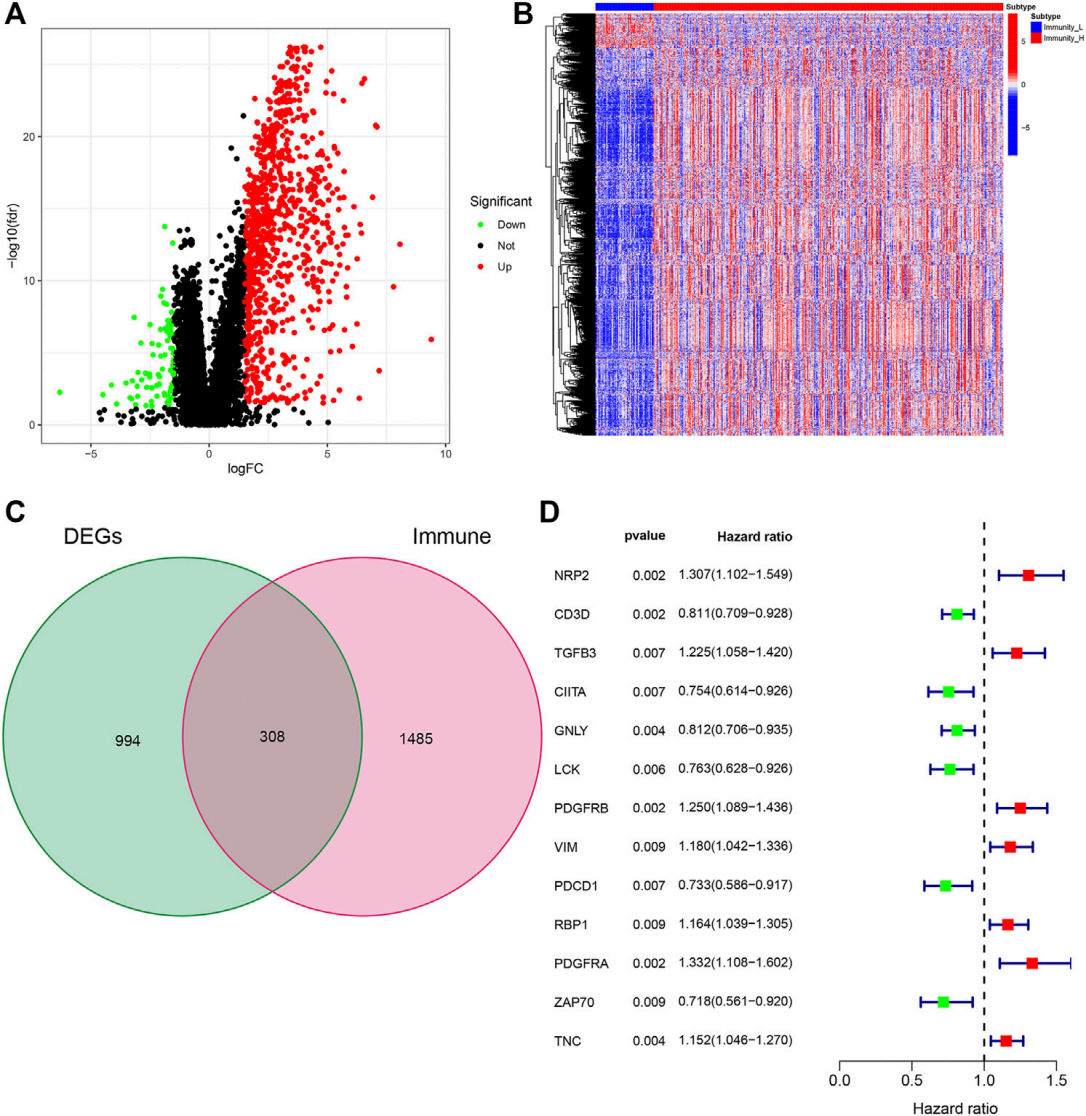
**(A)**Volcano plot of all differentially expressed genes (DEGs) showing the log2 (fold change) and FDR value of each gene. **(B)** DEGs expression between the two subtypes in the heat maps. **(C)**Veen plot based on the intersection of DEGs and human immune genes. **(D)** Forest plot based on univariable Cox proportional hazards regression analysis showing the prognosis-related immunity genes (PIMGs) and their hazard ratios.

We further studied the expression of differential genes of immune stratification in BLCA patients. The FDR values and log2 fold change multiples of the immune differential genes in the Immunity_H group and the Immunity_L group were showed in Figure 2A. After primarily screening, we totally identify 994 DEGs, of which 812 genes were up-regulated and 82 genes were down-regulated (Figure 2B). Subsequently, 308 DEGs were selected as DEIG using the ImmPort database (Figure 2C). Univariate Cox regression analysis indicated that 13 PIMGs had significant association with the survival of BLCA patients in DEIGs (*p* < 0.01), of which seven genes, including *NPR2, TGFB3, PDGFRB, PDGFRA, VIM, RBP1, RBP1* and *TNC*, increased the risk of prognosis, while the rest, including *CD3D, CIITA, GNLY, LCK, PDCD1* and *ZAP70,* were conducive to survival (Figure 2D).

### Identifying Prognosis-Related Genes and Constructing the Prognostic Model

**FIGURE 3:**
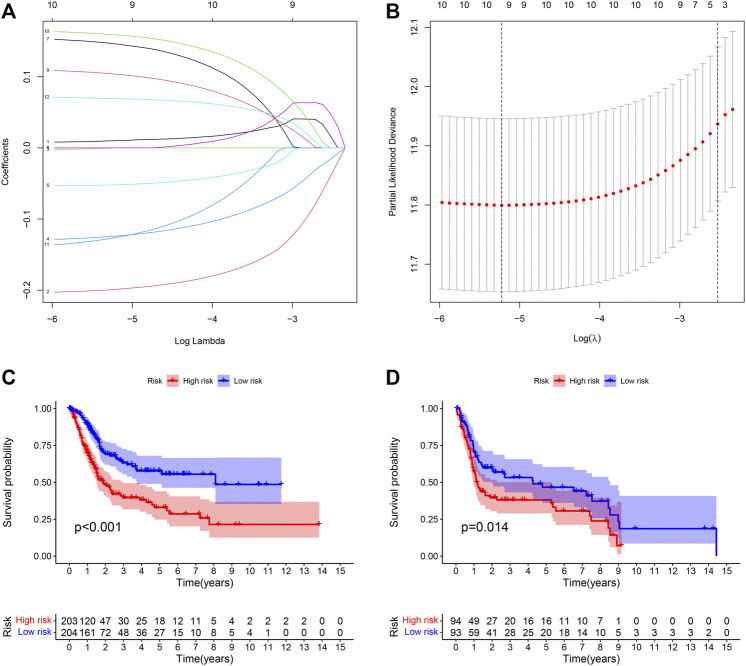
**(A)** LASSO coefficient curves were selected with simulation parameters set to 1000. **(B)** 10-fold cross-validation of selecting tuning parameter in the LASSO model. **(C)** Kaplan-Meier survival analysis of the PMB-based risk signature in the TCGA-BLCA cohort. **(D)** Kaplan-Meier survival analysis of the PMB-based risk signature in the GSECD cohort.

LASSO Cox regression analysis was performed on 13 selected PIMGs (Figures 3A,B). Finally, 9 LPIMGs were identified and their risk-correlation coefficients were calculated to determine the prognosis of BLCA patients. The risk score was calculated as follows: riskScore = NRP2*0.0101119 + CD3D*−0.1990949 + GNLY*−0.1241769 + LCK*−0.0519549 + VIM*0.1464182 + RBP1*0.1038418 + PDGFRA*0.1589969 + ZAP70*−0.12644895 + TNC*0.0693184. Data from TCGA was selected as the training group, the risk score of each BLCA case in this group was calculated, and all cases were classified into the high-risk group (203 patients) and the low-risk group (204 patients) based on the median risk score of 0.4886 (Supplementry Data S1; Supplementary Figure S1C). The correlation analysis indicated that the risk score had significant negative correlation with the survival time of BLCA patients which gradually decreased with the increase of the risk score (Supplementary Figures S1D,E). The Kaplan-meier curve showed that the difference in overall survival (OS) between the high-risk group and the low-risk group was significant, and patients in the low-risk group had a longer overall survival time than those in the high-risk group (Log-rank test, *p* < 0.0001) (Figure 3C). In order to evaluate the predictive power and accuracy of PMB-based risk characteristics, the ROC curves of the training group were drawn, and the AUC values of 1-, 3- and 5-year survival were 0.688, 0.719, and 0.706, respectively (Supplementary Figure S1F). The accuracy of the prognostic model was verified by the calibration chart, which suggested that the predicted value of the prognostic model was in good consistence with the actual value (Figure 3H). Besides, GSE31684 and GSE39281 were used as the external validation group, and we combined their data (GSECD) using R “sva” package to further confirm the accuracy and feasibility of the prognostic model, and the number of deaths in the high-risk group increased significantly (Supplementary Data S2). Then, the Pierce correlation analysis and Kaplan-Meier curves suggested that the constructed PMB-based risk characteristics still had good predictive power in the external validation group (Figure 3D).

### Combined Analysis of Tumor Immune Microenvironment and the Model of Prognosis

**FIGURE 4:**
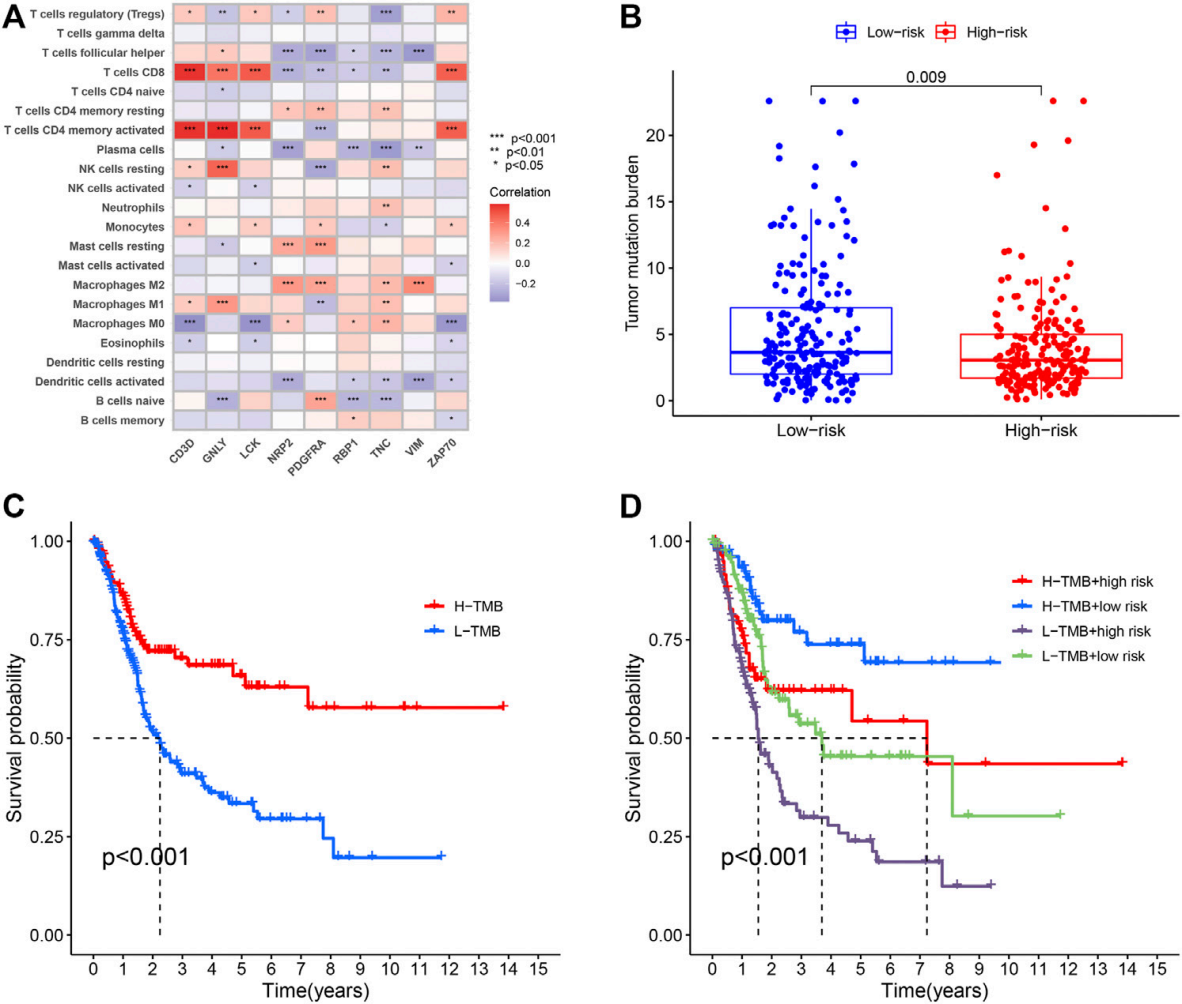
**(A)** Heatmap showing the correlation of the prognostic model of BLCA (PMB)-based risk signature with immune cell infiltration. The red suggesting the positive correlation while blue suggesting the negative correlation. **(B)** The comparison of TMB between High- and low-risk groups. **(C)** Kaplan-Meier survival analysis of the TMB in the TCGA-BLCA cohort. **(D)** Kaplan-Meier survival analysis of four groups stratified by combining the TMB and the PMB-based risk signature in the TCGA-BLCA cohort.

In order to investigate the correlation between immunotherapy and bladder cancer, 14 immune checkpoint inhibitors inlcuding *BTLA, GITR, TNFRSF14, IDO, LAG-3, PD-1, PD-L1, PD-L2, CD28, CD40, CD80, CD137, CD27*, and *Ctla-4* were selected for analysis. It was found that the risk score had negative correlation with the BTLA, CD27, CD40, CD80, and TNFRSF14 expression, which had significant differences in different risk groups (Supplementary Figure S2), indicating that tumor immunosuppression might lead to an increased risk score of patients. TYK2 and ACE2 were also differentially expressed in different risk groups, and with the increase of the risk score, their expression decreased (Figure 4A). In the TCGA-BLCA cohort, the TMB of patients in the high-risk group was significantly lower than that in the low-risk group (*p* = 0.009) (Figure 4B). In order to find the potential correlation between TMB and the prognosis of patients, according to the TMB cutoff value of 4.632, we divided the patients into the high TMB group, and the low TMB group (Supplementary Data S3). We found that the survival time of patients in the high TMB group was significantly lower than that of the low TMB group (*p* < 0.001) (Figure 4C). In order to evaluate the outcomes of patients more comprehensively, we investigated whether the combination of the risk score and TMB could be a more accurate prognostic marker. We integrated PMB-based risk characteristics with TMB, stratified all samples into the H-TMB/high risk, H-TMB/low risk, L-TMB/high risk, and L-TMB/low risk groups. Figure 4D suggested that differences between groups were significant (log-rank test, *p* < 0.0001), and in the H-TMB/low risk group, patients had the longest overall survival. The above results together suggested that the risk score had positive correlation with the degree of malignant tumor.

### Establishing a Nomogram With Clinical Features

**FIGURE 5:**
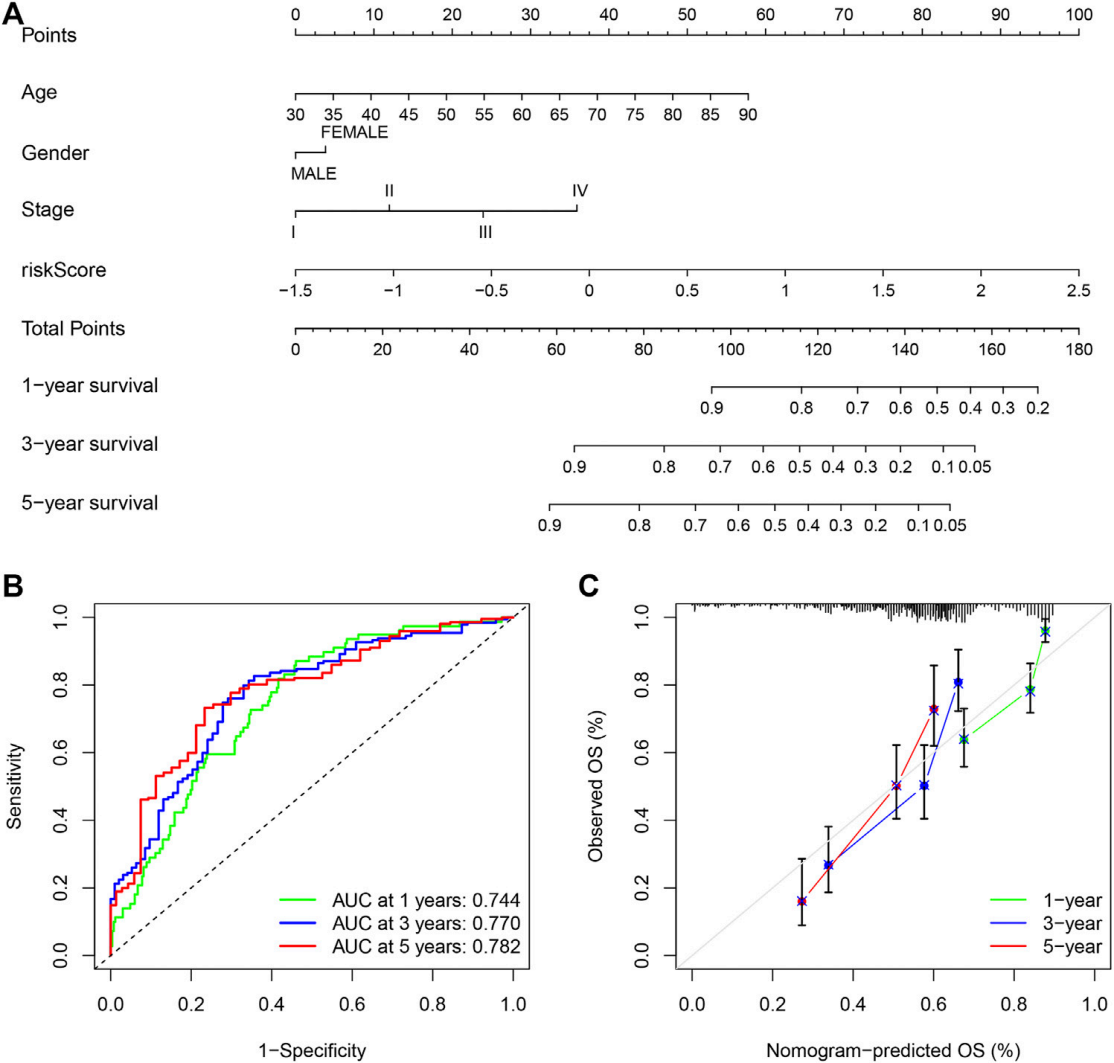
**(A)** Nomogram of age, gender, stage and risk score as independent prognostic factors for predicting overall survival. **(B)** The receiver operator characteristic (ROC) curves and the area under the curve (AUC) of the predictions for 1-, 3-, and 5-years of the nomogram for TCGA-BLCA cohort. **(C)** The calibration chart of the nomogram for TCGA-BLCA cohort.

Due to the significant correlation between the risk score and the degree of malignant tumor, univariate and multivariate Cox regression analysis for age, sex, and stage as covariates was conducted to test the potential possibility of the risk score as an independent prognostic factor for BLCA patients, of which the results showed that the PMB based risk characteristics had a *p* value less than 0.001, confirming that the PMB based risk characteristics could be used to predict the prognosis of BLCA patients (Table 1). Combined with the above factors, we constructed a nomogram (Figure 5A) to expand the clinical application and usability of PMB. The total score of each patient was obtained by calculating and summing the score for each prognostic parameter. The higher the total score was, the worse the patient’s clinical outcome was. The ROC curve showed that the nomogram had a good predictive ability for the survival rate, with a high accuracy, and the AUC values of 1-, 3-, and 5-year survival were 0.744, 0.770, and 0.782, respectively (Figure 5B). In addition, the calibration chart indicated that the nomogram performed similarly with the ideal model (Figure 5C).

**TABLE 1:** Univariable and multivariable Cox analysis of clinical characteristics and riskScore in the TCGA-BLCA cohort.

	Univariate cox regression				Multivariate cox regression			
ID	HR	HR.95L	HR.95H	pvalue	HR	HR.95L	HR.95H	pvalue
Age	1.039588391	1.022252149	1.057218636	6.04E-06	1.035655214	1.018448898	1.053152224	4.16E-05
Gender	0.913510834	0.6440517	1.29570661	0.611966738	0.870137802	0.611038106	1.239104053	0.440546516
Stage	1.822621822	1.479575308	2.245205288	1.68E-08	1.545728603	1.243151661	1.921951269	8.92E-05
riskScore	3.002207633	2.158508821	4.175683964	6.55E-11	2.483209078	1.749461945	3.524699316	3.58E-07

### Gene Set Pathway Enrichment Analysis

**FIGURE 6:**
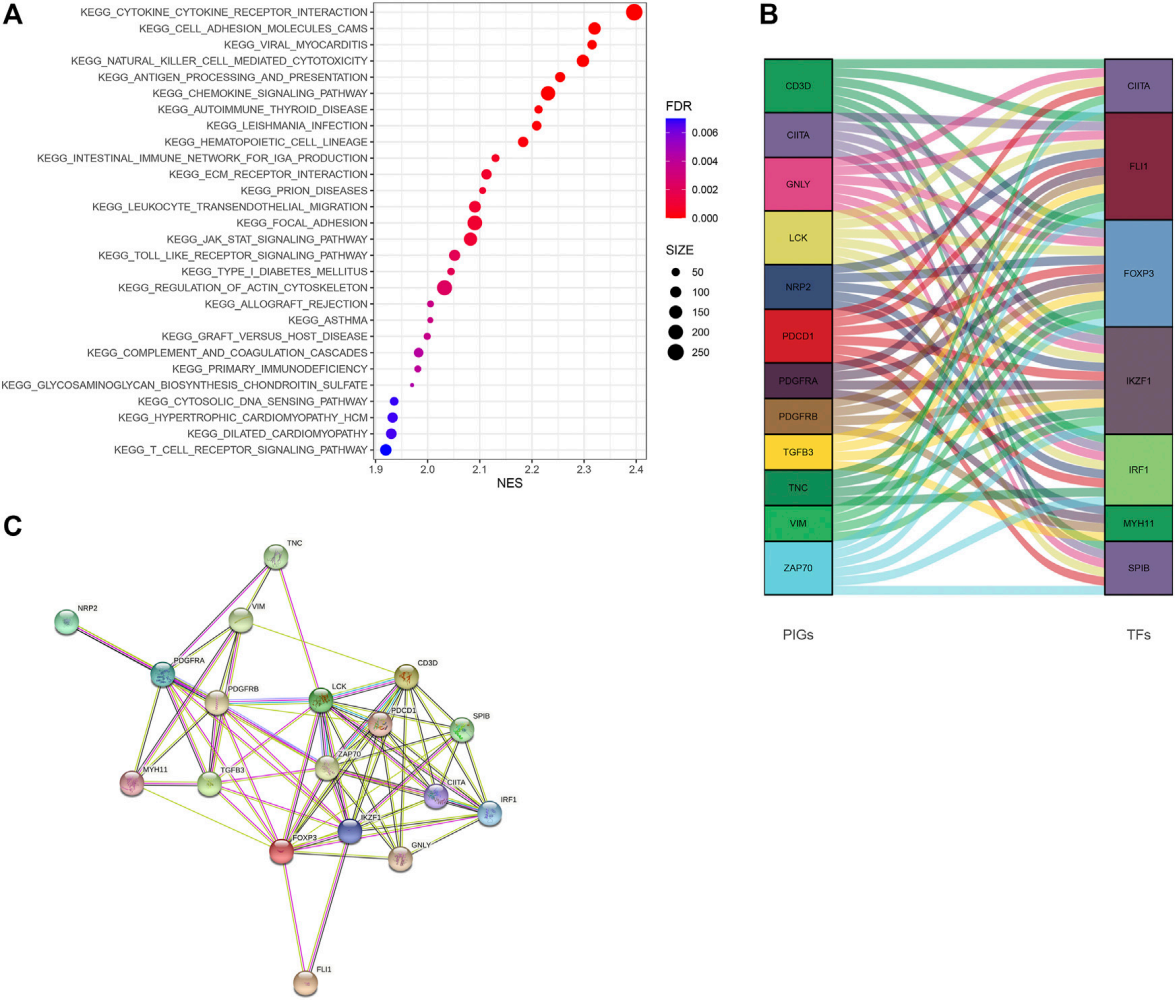
**(A)** The Kyoto Encyclopedia of Genes and Genomes (KEGG) enrichment analysis of DEGs. **(B)** Alluvial diagram of the BLCA TFs and PIMGs revealing their regulatory network. **(C)** PPI network between BLCA TFs and PIMGs. Network nodes representing proteins, and edges representing protein-protein associations, including both functional and physical protein associations. Line thickness indicating the strength of data support. The thicker line representing the higher confidence. KEGG, Kyoto Encyclopedia of Genes and Genomes; BLCA TF, Bladder cancer transcription factors; PIMGs, prognosis‐ associated immunity genes.

GSEA revealed that immune-associated pathways in the Immunity_H group were highly active, including the signaling pathway of T cell receptor, the pathway of antigen processing and presentation, cytokine involved immune response, and hematopoietic cell lineage. Additionally, various pathways of immune-associated disease were identified in the Immunity_H roup, including asthma, primary immune deficiency, graft-versus-host disease, allograft rejection, thyroid disease related to autoimmune, and immunity to leishmania infection (Figure 6A). In order to clarify the role of the multi-dimensional regulatory network of immune molecules in the occurrence and development of bladder cancer, we firstly explored the upstream mechanism of PIMG. By combining differential expression analysis with data from the CISTROME database, we identified transcription factors significantly associated with the BLCA prognosis. For the Immunity_H subtype, a total of 7 up-regulated transcription factors were identified. Figure 6B showed the regulatory network of BLCA TF-PIMGs. PPI analysis was further conducted and we confirmed the significant correlation between BLCA TF and PIMG (Figure 6C).

## Discussion

In our study, we collected gene expression data and clinical information of BLCA from the public databases. A total of 9 immune-related prognostic genes were identified by the Lasso analysis. Subsequently, a nine-gene prognostic model of BLCA (PMB) was established. We integrated clinical characteristics and risk scores to establish a nomogram. The ROC curve and calibration chart verified the prognostic accuracy of the nomogram. The high-risk KEGG analysis showed that the main functions of genes in the high-risk group were closely related to immunity. Finally, TMB had a significant correlation with the prognosis of patients, and had a potential connection with the PMB model. These findings strongly implied that immunity played a non-negligible role in the occurrence of BLCA.

We used Lasso regression to establish a PMB model, and used the file Supplementary Data S4, S5 and the code Supplementary Data S6 to achieve the repetition of the results of the model. Wu and Ma (2015) believed that in the researches of genetic analysis, most of the analyzed genes were expected to be “noise”, and only a few were related to the results and phenotypes. In the process of eliminating “noise” genes, a variety of machine learning methods (LASSO, adaptive LASSO, SCAD, and MCP) had been used. For the low-dimensional genomics data, stable approaches were widely developed, while for the high-dimensional genomics data, the development of approaches was limited. Therefore, in the process of screening genes, a variety of machine learning methods are worthy of our further trial and comparison. Ren et al. (Ren et al., 2019) believed that because gene expression might show heavy tailed distributions (especially for the high-expression genes), or be contaminated, the gene regulation relationship inference based on non-robust methods might be biased. Thus, we proposed a robust network based on the regularization and variable selection method for high-dimensional genomics data in cancer prognosis, and correspondingly also used “regnet” package in R language. The robust and regularized AFT model was fitted by the network penalty, and 9 prognostic genes were obtained by Lasso regression analysis. As we deeply understand the new machine learning methods, we will introduce new methods such as “regnet” at the design stage of bioinformatics analysis to explore more possibilities in the future.

In the past, some prognostic models of BLCA patients had been established (Dong et al., 2021; Liu et al., 2021), but in these studies, the tumor-immune-TMB interaction have not been fully considered. For the TCGA-BLCA patients, we firstly, based on immunogenomics analysis, divided the patients into the high immune (Immunity_H) subtype and the low immune (Immunity_L) subtype. Compared with the Immunity_L subtype, we found that the Immunity_H subtype showed stronger immune cell infiltration and higher expression of HLA genes, which suggested stronger immunogenicity. The Immunity_H subtype had abundant immune-related characteristics, and was rich in a lot of cancer-related pathways, such as leukemia, pancreatic cancer, and melanoma. What’s more, the results of our study found the potential association between immune activity and pathway activity for BLCA patients.

According to the expression of these 9 immune genes, the PMB based risk characteristics was developed, as a new predictive tool for the prognosis of BLCA, and was validated in the two data sets of GSE31684 and GSE39281. The results showed that the OS curves of patients with high- and low-risk scores were significantly different. Based on the risk characteristics of PMB combined with immune invasion, the prognosis of patients was predicted, and the survival time of patients in the low-risk/Immune-L group was the longest. Of the 9 genes used to construct the PMB, five oncogenes, namely *NRP2, VIM, RBP1, PDGFRA*, and *TNC*, were promising therapeutic targets. *NRP2* (Neuropilin 2) can regulate the activity of vascular endothelial growth factor-activated receptor, protein binding, and heparin binding, and take part in the positive regulation of angiogenesis, endothelial cell proliferation, cell adhesion, endothelial cell migration and other pathways, and its targeted drugs can treat hypoplasia in children (Estrada et al., 2021). *VIM* (Vimentin) is involved in the combination of double-stranded RNA, the formation of cytoskeleton, the formation of the lens of the eye, negative regulation of neuron projection development, astrocyte development, and cytokine-mediated signaling pathway. *RBP1* (Retinol-binding protein 1) is involved in several physiological functions (Gao et al., 2020), including regulation of metabolism and retinol transport. *PDGFRA* (platelet derived growth factor receptor alpha) mutations cause a variety of heterogeneous gastrointestinal mesenchymal tumors (Ricci et al., 2015), and *TKIs* inhibiting the most common driving mutations in *KIT* or *PDGFRA* might have brought about radical changes in treating gastrointestinal stromal tumors in the past 20 years (Zalcberg, 2021). *TNC* (enascin-C) is a large extracellular matrix glycoprotein that promotes cell adhesion and tissue remodeling, and is involved in the transduction of cellular signaling pathways (Spenlé et al., 2021). These findings encourage us to explore the molecular mechanisms of these genes in BLCA in the future.

It has been proved that immune checkpoint inhibitors, such as nivolumab, pembrolizumab, ipilimumab, atezolizumab, avelumab, and durvalumab, are effective for treating metastatic urological neoplasms (Petzold et al., 2021). We found that five immune checkpoint inhibitors, including CD27, CD40, CD80, BTLA, and TNFRSF14, were significantly negatively correlated with the risk score of patients, indicating that the risk of patients would increase with the increase of immune expression. Sensitivity to CD40 ligation-induced apoptosis might be a new mechanism to eliminate tumor transformation of urothelial cells. The important adaptive mechanism for the occurrence and development of transitional cell carcinoma might be CD40 expression loss (Bugajska et al., 2002). CD80 is an essential membrane antigen for the activation of T lymphocytes. CD80 monoclonal antibody inhibits the adjuvant stimulation of CD80, and prevents the differentiation of B lymphocytes into plasma cells, which plays a prominent role in the treatment of tumors (Vackova et al., 2021). CD27 and CD40 belong to the tumor necrosis factor receptor (TNFR) family. As a co-stimulatory pathway molecule, CD40 has been proven to be very successful in combination with pro-active drug antibody targets in both single-dose therapy and combination therapy (Peters et al., 2009). CD27 can stimulate the anti-tumor effect of monoclonal antibodies, and the stimulation of CD27 on the T cells surface and NK cells can increase the release of chemokines (Seidel et al., 2016). B- and T-lymphocyte attenuator (BTLA) is also known as B- and T-lymphocyte-associated protein. Under normal physiological conditions, the combination of BTLA and its ligand HVEM can inhibit the over-activation of lymphocytes *in vivo*, and prevent the immune system from damaging itself (Yu et al., 2021). Finally, TNFRSF14 might exert a tumor suppressor effect in bladder cancer by inducing cell apoptosis and inhibiting proliferation (Zhu and Lu, 2018). These immune-related studies are worthy of further exploration in the immunotherapy of bladder cancer in the future.

BLCA patients with a higher level of TMB had better prognosis, and when TMB increased, the response rate of immunotherapy was higher, implying that TMB might be an independent biomarker that can provide the guidance for more effective immunotherapy and improve the prognosis of BLCA (Ready et al., 2019). In addition, we observed that PMB was significantly correlated with TMB. Compared the AUC values of the ROC curves between the two groups, the combination of TMB and PMB also could predict the survival of patients. These findings suggested that risk characteristics based on PMB might help measure the responses to immunotherapy.

There are some limitation in our study. Firstly, the underlying mechanism of how the identified 9 LPIMGs regulate the BLCA process is still unclear, and their biological functions need to be further explored by experiments. Secondly, the development and verification of this model are only based on the public databases, and thus more clinical research data is still necessary to verify its effectiveness. Lastly, regarding the machine learning methods, we used Lasso regression to perform the gene screening and completed all the research, but Lasso regression may not be the most ideal method to identify relevant features (such as gene expression). The new method of “regnet” are worthy to use in the future study.

## Conclusion

In summary, we had identified nine genes, including *PDGFRA, VIM, RBP1, RBP1*, *TNC*, *CD3D, GNLY, LCK,* and *ZAP70*, which played important roles in the occurrence and development of BLCA. The prognostic model based on these genes had good accuracy in predicting the OS of patients and might be promising candidates of therapeutic targets. In addition, further experimental studies are necessary to reveal the underlying mechanisms by which these genes mediate the progression of BLCA.

## Data Availability

The datasets presented in this study can be found in online repositories. The names of the repository/repositories and accession number(s) can be found in the article/Supplementary Material.

## Supplementary Material

Supplementry Figure 1
**(A)** Validation of immunophenotype via tSNE; **(B)** The comparison of the immune cell infiltration level between two subtypes; **(C,E)** Distributions of the risk score, survival time, and survival status in the TCGA-BLCA cohort; **(D)** Correlation analysis of the risk score and survival time in the TCGA-BLCA cohort; **(F)** The ROC curves and AUC of the predictions for 1, 3, and 5 years of the PMB-based risk signature for TCGA-BLCA cohort.

Supplementary Figure 2 Boxplots and Scatter plots depicting correlation between the PMB-based risk signature and gene expression of immune checkpoint inhibitors. **(A,B)** BTLA; **(C,D)** CD27; **(E,F)** CD40; **(G,H)** CD80; **(I,J)** TNFRSF14.

Supplementary Data 1 Distributions of the risk score, survival time, and survival status in the TCGA-BLCA cohort.

Supplementary Data 2 Gene expression, risk score and risk stratification of patients in the GEO database.

Supplementary Data 3 Dividing TCGA-BLCA patients into different groups based on TMB

Supplementary Data 4 Gene expression significantly associated with the patient’s prognosis in the TCGA-BLCA cohort Supplement data

Supplementary Data 5 The expression of genes significantly associated with the patient’s prognosis in the GEO database.

Supplementary Data 6 The code of Lasso regression analysis (R 4.0.5).
