# Supplementary figures and images for: Identification of Immune-Related Genes Associated With Bladder Cancer Based on Immunological Characteristics and Their Correlation With the Prognosis
Source: Front Genet. 2021 Nov 26;12:763590. doi: 10.3389/fgene.2021.763590 (PMC8664377; doi:10.3389/fgene.2021.763590)

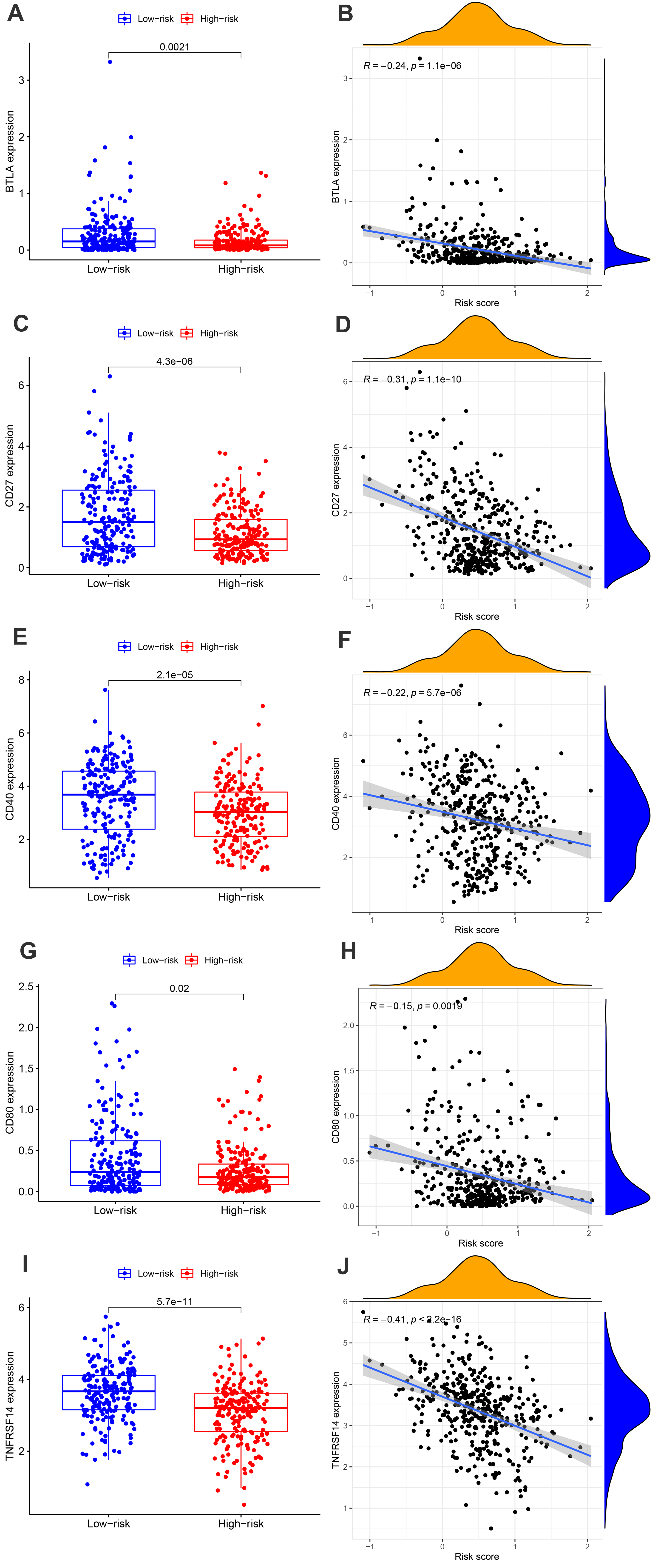

Supplement: Supplementary file 2 [file Image2.TIF]

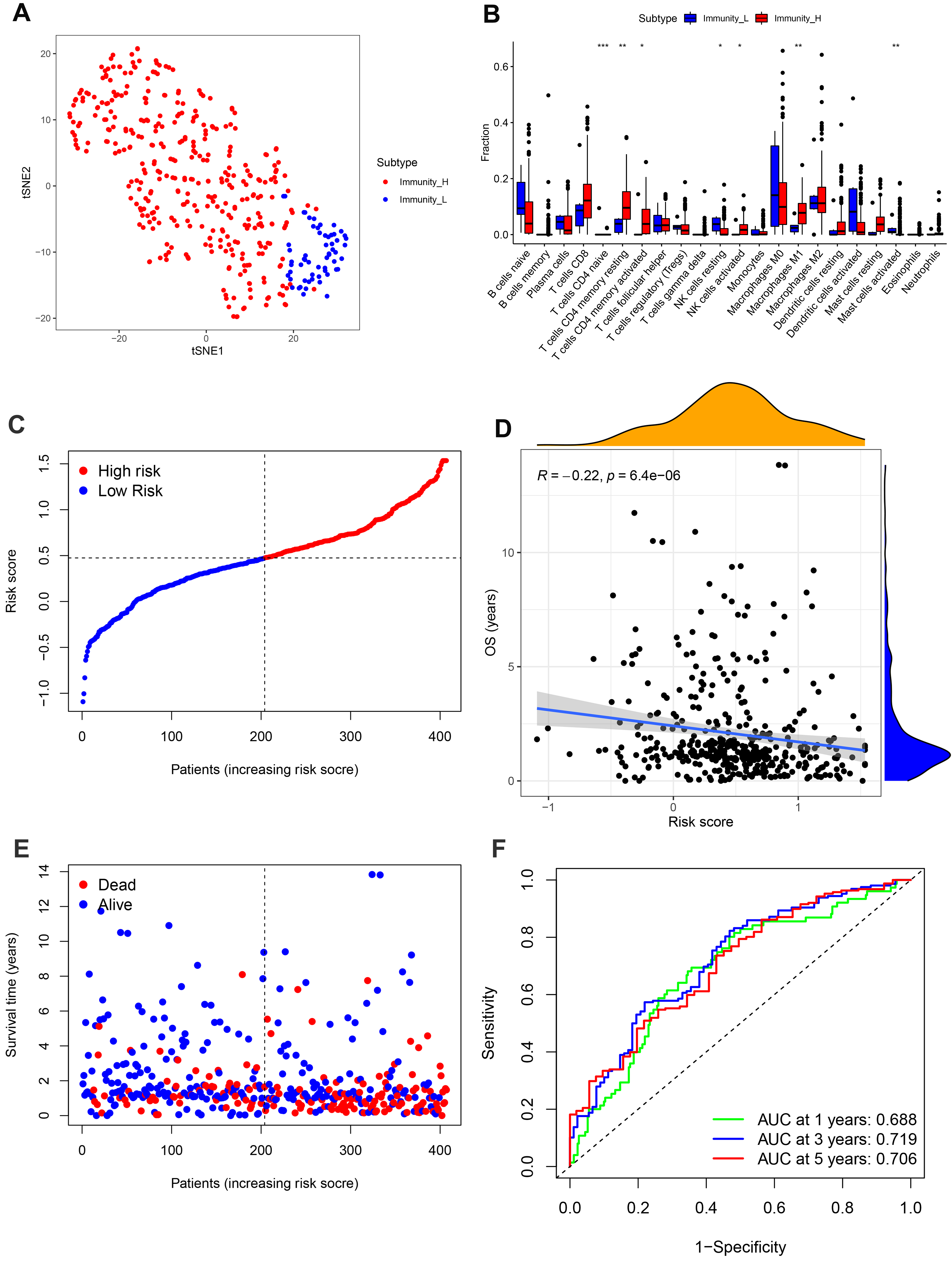

Supplement: Supplementary file 3 [file Image1.TIF]
